# Treatment with Pyranopyran-1, 8-Dione Attenuates Airway Responses in Cockroach Allergen Sensitized Asthma in Mice

**DOI:** 10.1371/journal.pone.0087558

**Published:** 2014-01-29

**Authors:** Soojin Park, Min-Sun Park, Kyung-Hwa Jung, Joohyun Song, You Ah Kim, Hi Jae Cho, Byung-Il Min, Hyunsu Bae

**Affiliations:** 1 College of Korean Medicine, Kyung Hee University, Seoul, Republic of Korea; 2 Department of East-West Medicine, Kyung Hee University, Seoul, Republic of Korea; 3 Natural Products Department, Korea Promotion Institute for Traditional Medical Industry, Kyungbuk, Republic of Korea; French National Centre for Scientific Research, France

## Abstract

Chronic allergic asthma is characterized by Th2-typed inflammation, and contributes to airway remodeling and the deterioration of lung function. Viticis Fructus (VF) has long been used in China and Korea as a traditional herbal remedy for treating various inflammatory diseases. Previously, we have isolated a novel phytochemical, pyranopyran-1, 8-dione (PPY), from VF. This study was conducted to evaluate the ability of PPY to prevent airway inflammation and to attenuate airway responses in a cockroach allergen-induced asthma model in mice. The mice sensitized to and challenged with cockroach allergen were treated with oral administration of PPY. The infiltration of total cells, eosinophils and lymphocytes into the BAL fluid was significantly inhibited in cockroach allergen-induced asthma mice treated with PPY (1, 2, or 10 mg/kg). Th2 cytokines and chemokine, such as IL-4, IL-5, IL-13 and eotaxin in BAL fluid were also reduced to normal levels following treatment with PPY. In addition, the levels of IgE were also markedly suppressed after PPY treatment. Histopathological examination demonstrated that PPY substantially inhibited eosinophil infiltration into the airway, goblet cell hyperplasia and smooth muscle hypertrophy. Taken together, these results demonstrate that PPY possesses a potent efficacy on controlling allergic asthma response such as airway inflammation and remodeling.

## Introduction

Asthma is a chronic inflammatory disease of the lungs that is characterized by airway inflammation, airway hyper-responsiveness (AHR) and airflow obstruction [1], [2]. In susceptible individuals, airway inflammatory disease causes recurrent episodes of wheezing, breathlessness, chest tightness and coughing, especially at night and early in the morning. These episodes are usually associated with a variable amount of airflow obstruction that is often reversible. Allergic airway inflammation also causes an associated increase in the existing bronchial hyper-responsiveness to a variety of stimuli.

Many types of cells play important roles in the development of bronchial asthma, including T lymphocytes, eosinophils, macrophages, neutrophils, mast cells and epithelial cells [3]–[5]. Th2 cells play a central role and control the allergic response through the production of cytokines such as interleukin (IL)-4, IL-5, and IL-13 [6]–[8]. IL-4 plays an important role in eosinophil infiltration into lung tissues, B cell maturation, and IgE synthesis. IL-5 is involved in the proliferation and maturation of eosinophils. IL-13 plays a role in eosinophilic inflammation, mucus hypersecretion, and AHR [2], [6], [8]–[10]. Eosinophils can affect the structural changes that occur in the airways of asthma patients, including goblet cell hyperplasia, enlarged submucosal mucus glands, and airway smooth muscle hypertrophy.

Eotaxin (CCL11) is an important eosinophil-specific chemokine that is released in the respiratory epithelium following allergic irritation [11], [12]. Conventional remedies, including corticosteroids and β_2_-agonists, are effective in managing asthma. However, there are concerns regarding the adverse effects and low curative efficacy of current remedies, which limit their long term usage [13]–[15]. These limitations mean that it would be beneficial to find alternative agents that have lower side effects and possess potent efficacy for treating asthma. Many natural products used in traditional medicine have often been found to be good candidates for the treatment of asthma [16], [17]. The results of our previous studies suggested that Moutan Cortex Radicis reduced eotaxin secretion [18] and that Schizandrae Fructus inhibited the migration of eosinophils to the lungs in a murine asthma model [19]. However, the biomolecular activity of many natural products has yet to be examined by extensive experimental testing_ENREF_1 [20]. Additionally, despite their remarkable curative abilities, most natural products have not been widely used in western societies because little is known about their mode of action at the molecular level.

Viticis Fructus (VF), which is the dried fruit of *Vitex rotundifolia* L. (*Vitex trifolia* L. var. *simplicifolia* Cham.), has long been widely used as a traditional herbal medicine in various Asian countries for the treatment of migraine pain, various allergic diseases and upper respiratory infections [21]. Pyranopyran-1, 8-dione (PPY) was isolated as a pale yellow powder from the Viticis Fructus extract. Intraperitoneal administration of PPY exerted an anti-asthmatic effect by inhibiting the development of pulmonary eosinophilic inflammation in an ovalbumin induced asthma model [22].

In this study, we investigated the attenuation of airway responses and the anti-inflammatory effects of orally administered PPY. We measured the effects of PPY in regards to airway function, antigen-induced inflammatory infiltrates in the airways, Th2 cytokine and eotaxin production, serum immunoglobulin levels, and histopathological changes of lung tissues in a mouse model of cockroach allergen induced airway responses.

## Materials and Methods

### Animals

The experiments were performed in accordance with the approved animal protocols and guidelines established by Kyung Hee University. The protocol was approved by Institutional Animal Care and Use Committee (KHUASP (SE) – 11 - 025) in Kyung Hee University College of Korean Medicine (Seoul, Korea). The experiments were conducted with 6 week-old (wt. 21∼22 g) mice. For the asthma experiments, Balb/c male mice were purchased from Charles River Korea (Seungnam, South Korea). All mice were kept under pathogen-free conditions with air conditioning and a 12-h light/dark cycle. In addition, all mice had free access to food and water during the experiments.

### Reagents

Pyranopyran-1, 8-dione (PPY), isolated from Vitex rotundifolia L. was provided from the Institute for Korea Traditional Medical Industry (Kyungsan, Kyungbuk, South Korea).

The fruits of Vitex rotundifolia L. (3 kg) were extracted with MeOH (5×10 L) for 24 h by percolation. The solvent was evaporated in vacuo to afford MeOH extract (100 g), which was then suspended in water (1 L), and partitioned with n-hexane (5×1 L), EtOAc (5×1 L), and n-BuOH (3×1 L), sequentially. The EtOAc extract (17 g) was separated by silica gel column chromatography (φ 4.5 cm; silica gel 70–230 mesh, 500 g) using gradient mixtures of MeOH in CHCl3 (0→50%, and washed with MeOH 100%, 5 L each) as mobile phases, affording 1H, 8H-Pyrano [3, 4-c] pyran-1, 8-dione (235 mg, 0.0078% w/w). PPY is a pale yellow powder that corresponds to the elemental formula C_8_H_4_O_4_. The purity of PPY was over 95% according to the HPLC analysis.

The concentration of CKA extract supplied by the manufacturers is expressed in weight to volume (w/v) units. A designation of 1∶10 w/v indicates that the solution contains the extractable material from 1 g of raw material added to 10 ml of buffer solution [23]. The level of endotoxin which is used synonymously with the term “lipopolysaccharide (LPS)” was 6,580 EU/ml. The concentrations of Bla g 1 and Bla g 2 in the extract were 429 U/ml and 129 ng/ml, respectively.

### Experimental Protocol and Design in the allergic asthma murine model

**Figure 1:**
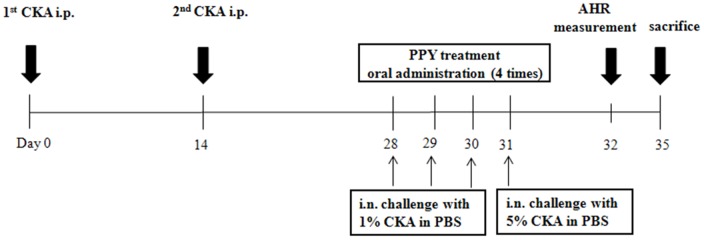
Schematic diagram of the experimental protocol. CKA-induced asthmatic groups were separated into 5 groups (CKA group, MK group, PPY 1 mg/kg group, PPY 2 mg/kg group and PPY 10 mg/kg group) (n = 6/group). Sensitization: CKA via i.p. injection (day 0, 14). Challenge: CKA via i.n. (day 28 to 31). PPY treatment: Oral gavage 2 hr before the CKA challenge. AHR was measured 24 hr after the last CKA challenges (day 32). BAL fluid, blood and lung tissues were collected (day 35).

The experiment was performed as described in Choi, Park *et al* and Holden T. Maecker *et al* [24], [25]. Briefly, the mice were sensitized by intraperitoneal (i.p.) injections with 10 µg of cockroach allergen (CKA) (Hollister-Stier, Spokane, WA, U.S.A.) in incomplete Freund's adjuvant (Sigma-Aldrich, St. Louis, MO, U.S.A.) on day 0 and 14 [26]. Subsequently, mice received an intranasal (i.n.) challenge with cockroach allergen (1% CKA in phosphate-buffered saline [PBS]) on day 28 to 30 and 5% CKA in PBS on day 31 (Figure 1). The experiment schedule was modified from the methods of McGee HS *et al* [27]. CKA-sensitized mice were treated with PPY (1, 2, 10 mg/kg/day) suspended in PBS by oral gavage 2 hr before the CKA challenge [28]. Negative control (NC) and CKA-exposed (CKA) groups were treated with only PBS by oral gavage, and the positive control (MK) group was treated with Montelukast sodium (10 mg/kg/day) suspended in PBS by oral gavage before the CKA challenge. In this experiment, for minimizing stress to mice, skillful and experienced researchers performed the oral injection with flexible plastic needle made for animal feeding (Cadence, Staunton, VA, U.S.A). The mice were analyzed using non-invasive lung function measurements (All Medicus, Seoul, Korea) to assess AHR. On day 35, mice were euthanized by intraperitoneal injection of pentobarbital sodium (50 mg/kg, Hanlim Pharm. Co., Seoul, Korea) and exsanguination without any previous intervention. The animal experiments were reported following guidelines recommended from the Animal Research: Reporting In Vivo Experiments (ARRIVE) (http://www.nc3rs.org.uk/page.asp?id=1357).

We performed experiments three times independently to clarify that PPY has significant effect on attenuation of cockroach allergen induced airway responses.

### Bronchoalveolar Lavage Fluid (BALF)

BAL fluid was collected by infusion and extraction of 1 ml of ice cold PBS. This procedure was repeated three times, and the lavages were pooled. Recovered BAL fluid (70–80%) was centrifuged at 1,300 rpm for 10 minutes. The cell pellets were resuspended in 1 ml PBS and adhered to glass slides using cytocentrifugation. Total viable cell counts were determined in a hemocytometer using trypan blue exclusion. Differential counts of eosinophils, neutrophils, lymphocytes, and macrophages were determined on Diff-Quick stained (Life Technologies, Auckland, New Zealand) cytospin smears of BAL fluid samples (5×10^5^/200 µl cells) from individual mice. BAL fluid was then centrifuged and the supernatants were kept at −80°C. The results are expressed as total cell number ×10^4^.

### Assessment of cytokines in BALF using Enzyme-Linked Immunosorbent Assay (ELISA)

The concentration of Th2 cytokines and eotaxin was measured with a quantitative sandwich enzyme-linked immunoassay kit (BD, San Diego, CA, U.S.A. for IL-4, IL-5 and R&D, Minneapolis, MN, U.S.A. for IL-13, eotaxin). 96-well immune-microplates (Costar, Corning, NY, U.S.A.) were incubated overnight at 4°C with anti-mouse IL-4, IL-5, IL-13 or eotaxin monoclonal antibodies in coating buffer, washed with PBS containing 0.05% Tween 20 (Sigma-Aldrich Co., St. Louis, MO, U.S.A.) and blocked with 5% FBS and 1% BSA in PBS. Subsequently, wells were loaded with 100 µl of BAL fluids and incubated for 2 hours at room temperature. The secondary peroxidase labeled biotinylated anti-mouse IL-4, IL-5, IL-13 or eotaxin monoclonal antibody in assay diluents for 1 hr. Finally, the plates were treated with TMB substrate solution (KPL, San Diego, CA, U.S.A.) for 30 minutes and the reaction was stopped by the addition TMB stop solution. Optical density was measured at 450 nm in a microplate reader (SOFT max PRO, version 3.1 software, Sunnyvale, CA, U.S.A). All results were normalized to the total BALF protein amount in each sample [29], [30].

### Determination of IgE titers using ELISA

For serum, a 96-well immuno-microplate (Costar, Corning, NY, U.S.A.) was coated with anti-mouse IgE monoclonal antibodies. Serum was diluted with 5% FBS in PBS (assay diluent) by 1∶250 and IgE was measured using a standardized sandwich ELISA, according to the manufacturer's protocol (BD). Optical density was measured at 450 nm in a microplate reader (SOFT max PRO, version 3.1 software, Sunnyvale, CA, U.S.A.).

### Histological examination of lung tissues

The lung tissues were removed from the mice. The right, lower lobes of the lung were removed for histological analysis. Four percent paraformaldehyde fixing solution was infused into the lungs. The specimens were dehydrated and embedded in paraffin. For histological examination, 4 µm sections of embedded tissue were cut on a rotary microtome, placed on glass slides, deparaffinized, and stained sequentially with hematoxylin and eosin (H&E). The severity of peribronchial inflammation was graded semi-quantitatively as previously described. Periodic acid-Schiff (PAS)-stained lung sections were examined at magnification of 400×. We quantified histologic images as previously described in Lee SH et al, Royce SG et al, Locke NR et al [31]–[33]. Lung tissues were stained with PAS in order to demonstrate the presence of mucin within goblet cells. The number of goblet cells within the bronchial epithelium was quantified as the percentage of PAS-positive cells. Four bronchioles randomly selected from each section of mouse lung tissue were used to analyze, and the mean goblet cell coverage of each section (%) was also calculated (Scale bar  = 20 µm) [34], [35]. The diameters of bronchi and bronchioles with goblet cell metaplasia were determined by using an Olympus BX51 microscope (Olympus, Tokyo, Japan) equipped with a DP71 digital camera (Olympus, Tokyo, Japan). Statistical analysis was calculated using the one-way ANOVA. In all cases, *p*<0.05 was considered to be statistically significant. All statistical analyses were carried out using the Prism 5 software (GraphPad Software Inc, Irvine, CA, U.S.A.). For immunohistochemistry (IHC) detection of smooth muscle myosin light chain 2 (MLC-2), 4 µm sections of the lower trachea and lung were treated with 0.3% H_2_O-methanol for 20 minutes to block endogenous peroxidases. The sections were then incubated at 4°C overnight with anti-MLC rabbit polyclonal antibody (1∶50 dilution; Santa Cruz Biotechnology, SantaCruz, CA, USA). After the slides were incubated with an avidin–biotin peroxidase complex (ABC kit, Vector Laboratories, Burlingame, CA, USA), the color was developed using 3, 30-diaminobenzidine tetrachloride (DAB; Zymed Laboratories, South San Francisco, CA, U.S.A.). After immunohistochemical staining, the slides were counterstained with Harris's hematoxylin for 1 min and mounted with Canada balsam (Show Chemical Co., Ltd., Tokyo, Japan). The areas of bronchial smooth muscle were measured using Image-Pro Plus software 6.1 (Media Cybernetics, Warrendale, PA, U.S.A) at magnifications of 200× [34], [36], [37].

### Measuring airway hyper-responsiveness (AHR) to methacholine

Non-invasive measurement of airway responsiveness was used in this study (All Medicus, Seoul, South Korea). Airway responsiveness was assessed in unrestrained and conscious mice at 24 hours after last challenge with CKA, as described in other studies [38]–[40]. Mice were placed in a barometric plethysmographic chamber (All Medicus, Seoul, Korea) and baseline readings were taken for 3 min. The enhanced pause (Penh) was calculated according to the following formula: [(expiratory time/relaxation time − 1) × (peak expiatory flow/peak inspiratory flow)]. Penh, which was calculated as according to the manufacturers' protocol, is a dimensionless value that represents a function of the proportion of maximal expiratory to maximal inspiratory box pressure signals. Penh was used as a measure of airway resistance to methacholine. Results were expressed as the percentage increase in Penh after challenge with each concentration of methacholine, where the baseline Penh value (after saline challenge) was expressed as 100%. Penh values averaged for 3 minutes after each nebulization were evaluated. The results were expressed as the relative increase in Penh following challenge with each concentration of methacholine (0, 25, 50 and 100 mg/ml).

### Statistical analysis

Statistical analysis of the data was conducted using the Prism 5 software (GraphPad Software Inc, Irvine, CA, U.S.A.). All values were presented as the means ± S.E.M (standard error of the mean). Differences between the means of the control and the treatment samples were determined by one-way ANOVA or Student's *t* test. In all cases, *p*<0.05 was considered to be statistically significant.

## Results

### PPY had an inhibitory effect on airway hyper-responsiveness (AHR)

**Figure 2:**
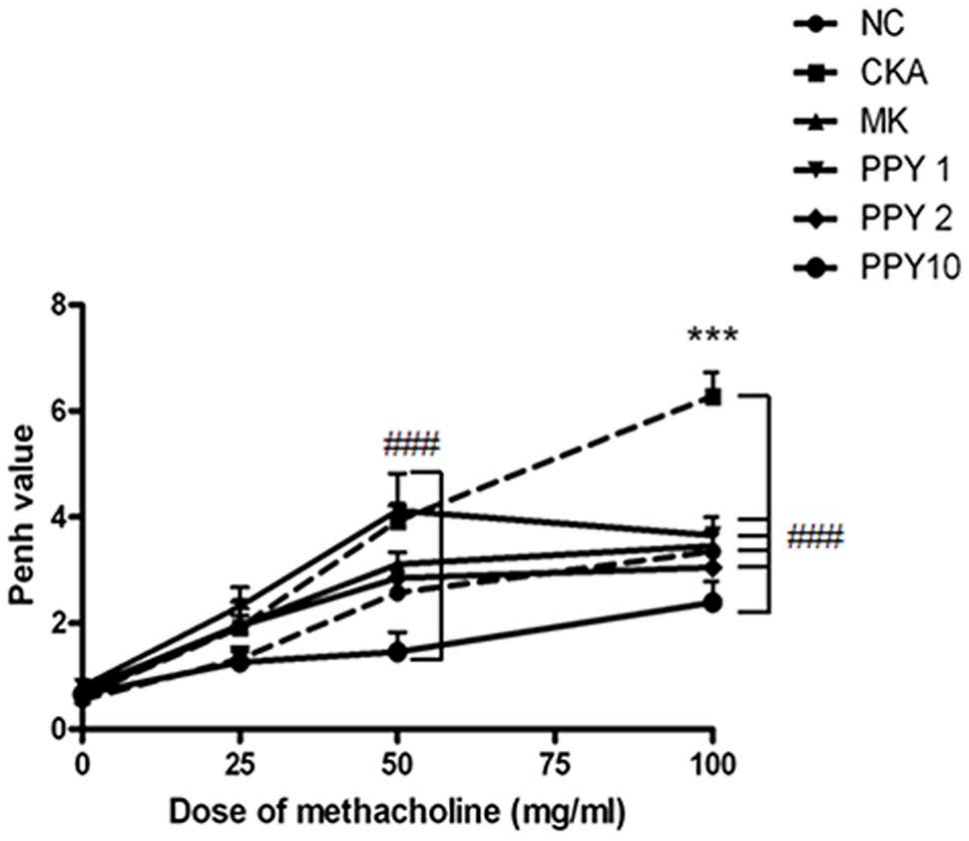
The treatment of allergic mice with PPY alleviates development of airway hyper-responsiveness (AHR). Twenty-four hours after the final intranasal CKA or PBS administration, to evaluate the inhibitory effect of PPY on airway hyper-responsiveness, whole body barometric plethysmographic analysis was performed. The mice were stimulated with increasing does of aerosolized methacholine (0, 25, 50 and 100 mg/ml). Normal control mice treated with PBS only (NC), CKA-challenged mice treated with PBS (CKA), CKA-challenged mice treated with 10 mg/kg Montelukast (MK), CKA-challenged mice treated with 1 mg/kg PPY (PPY 1), CKA-challenged mice treated with 2 mg/kg PPY (PPY 2) and CKA-challenged mice treated with 10 mg/kg PPY (PPY 10).The data are represented as the mean ± S.E.M. of 6 mice. Statistical analysis was conducted by one-way ANOVA followed by the Newman-Keuls Multiple Comparison test (significantly different from NC, *P<0.05, **P<0.01, ***P<0.001; significantly different from CKA, *#P*<0.05, ##*P*<0.01, ###*P<*0.001, n = 6).

Twenty-four hours after the final intranasal CKA or PBS administration, to evaluate the inhibitory effect of PPY on airway hyper-responsiveness, whole body barometric plethysmographic analysis was performed. The Penh value of the CKA-exposed mice was significantly higher than of the PBS-treated NC group at 100 mg/ml (6.2±0.4 *vs* 3.3±0.3) of methacholine. Treatment with 10 mg/kg of PPY significantly reduced the increased Penh values of the CKA-induced mice at 50 mg/ml of methacholine, similar to the level of the NC group. The PPY (1, 2, 10 mg/kg) treatment significantly reduced the increased Penh values of the CKA-induced mice at 100 mg/ml methacholine, similar to the level of the NC group. Montelukast treated mice showed an AHR decrease similar to that achieved using PPY (Figure 2).

### Alteration of pulmonary cytokine and chemokine levels with PPY

**Figure 3:**
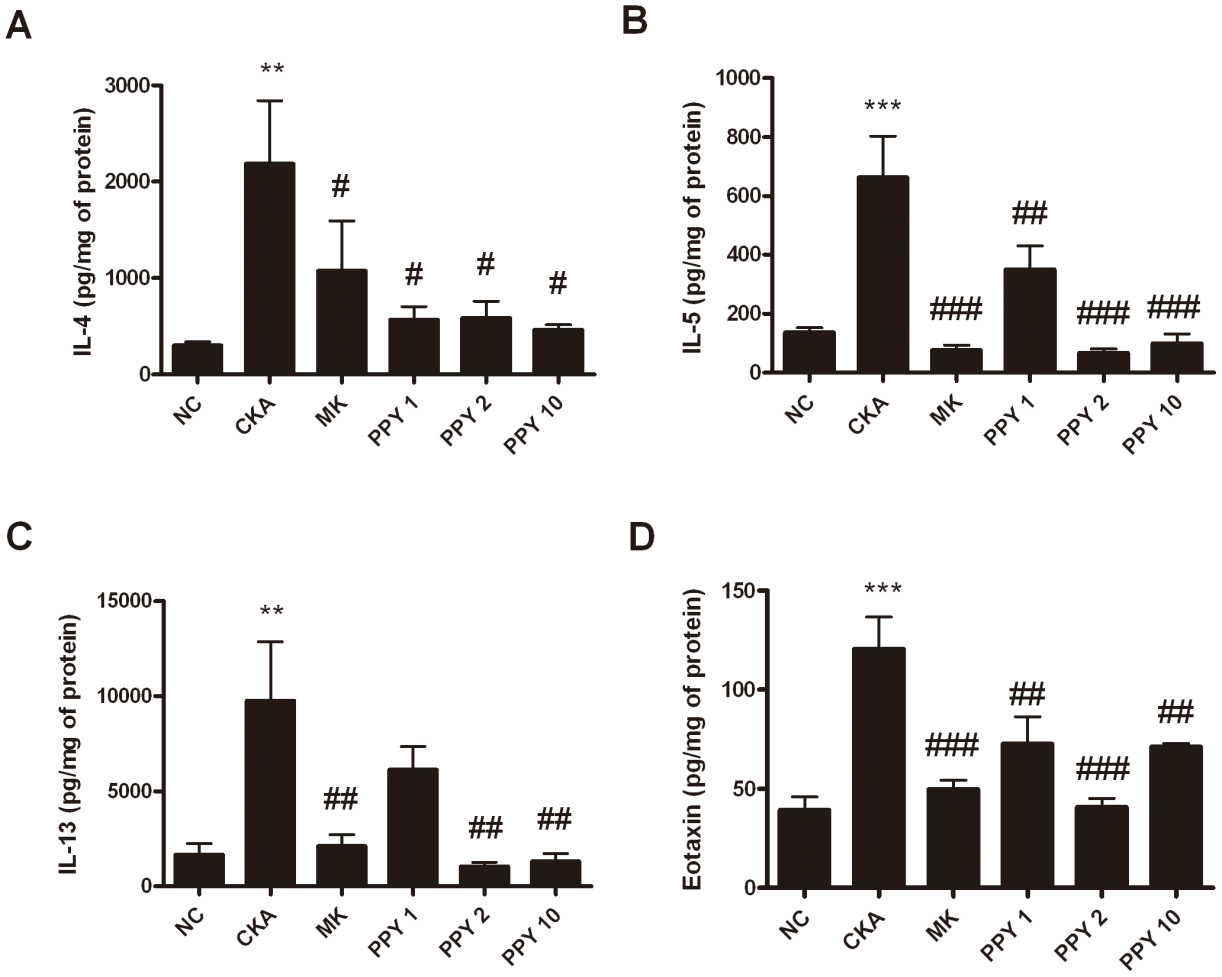
PPY reduces the level of Th2 cytokines and eotaxin in BAL fluid. A) IL-4 level, B) IL-5 level, C) IL-13 level and D) Eotaxin (CCL11) level. BAL fluid was collected by infusion and extraction of ice cold PBS. The concentrations of IL-4, IL-5, IL-13 and Eotaxin (CCL11) were measured with a quantitative sandwich enzyme-linked immunoassay kit. Expression of 4 different targets was analyzed in 100 µl of BAL fluid and normalized to the total protein amount in each sample. Normal control mice treated with PBS only (NC), CKA-challenged mice treated with PBS (CKA), CKA-challenged mice treated with 10 mg/kg of Montelukast (MK), CKA-challenged mice treated with 1 mg/kg of PPY (PPY 1), CKA-challenged mice treated with 2 mg/kg of PPY (PPY 2) and CKA-challenged mice treated with 10 mg/kg of PPY (PPY 10).The data are shown as the mean ± S.E.M. Statistical analysis was conducted by one-way ANOVA followed by the Newman-Keuls Multiple Comparison test (significantly different from NC, *P<0.05, **P<0.01, ***P<0.001; significantly different from CKA, *#P*<0.05, ##*P*<0.01, ###*P<*0.001, n = 6).

To further characterize the nature of the response to PPY treatment, additional studies were designed to examine an oral dose response (1, 2, 10 mg/kg). CKA challenge caused a significant increase in BAL fluid concentrations of IL-4, IL-5, IL-13 and eotaxin (CCL11) compared to the NC group. As shown in Figure 3(A, B, C, D), IL-4, IL-5, IL-13 and eotaxin levels were significantly reduced in PPY-treated (1, 2, 10 mg/kg) mice. In addition, treatment with 2 and 10 mg/kg of PPY groups were similar to the NC group on IL-13. However, treatment with 1 mg/kg of PPY group did not show significant decrease of IL-13 levels no more than CKA-exposed group. Also, IL-5 and IL-13 levels observed treatment with 10 mg/kg of PPY remarkable reduced compared with 1 mg/kg of PPY. The results of this study suggested that treatment with 10 mg/kg of PPY shows effects on CKA induced allergic asthma in mice better than 1 mg/kg of PPY (Figure 3).

### Inhibition of serum IgE production

**Figure 4:**
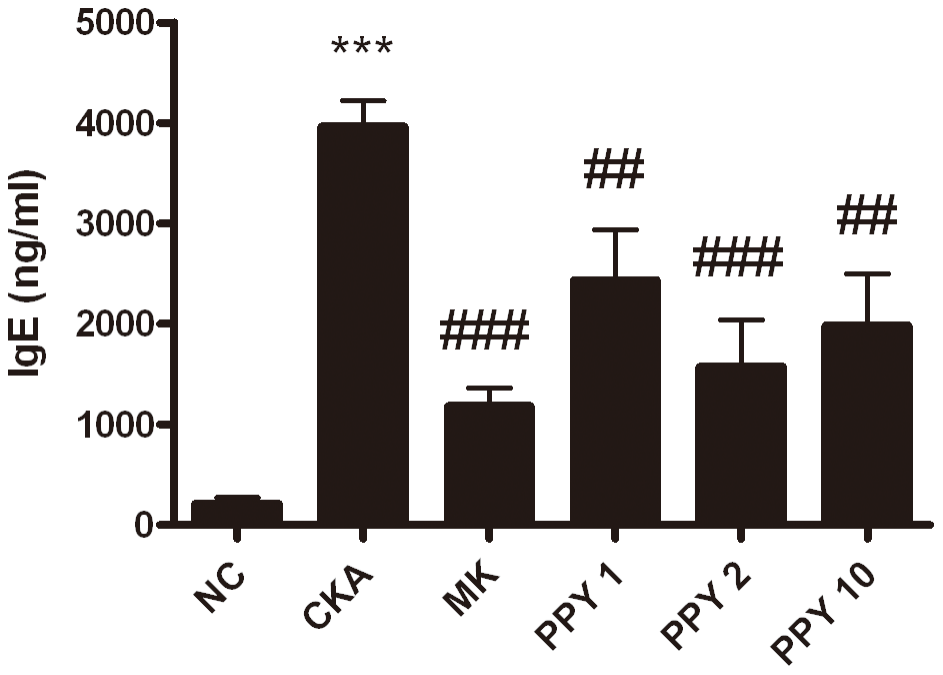
The concentration of total IgE in the serum. Blood was collected from the retro-orbital plexus. The amount of IgE was evaluated by ELISA. Normal control mice treated with PBS only (NC), CKA-challenged mice treated with PBS (CKA), CKA-challenged mice treated with 10 mg/kg of Montelukast (MK), CKA-challenged mice treated with 1 mg/kg of PPY (PPY 1), CKA-challenged mice treated with 2 mg/kg of PPY (PPY 2) and CKA-challenged mice treated with 10 mg/kg of PPY (PPY 10). The data are shown as the mean ± S.E.M. Statistical analysis was conducted by one-way ANOVA followed by the Newman-Keuls Multiple Comparison test (significantly different from NC, *P<0.05, **P<0.01, ***P<0.001; significantly different from CKA, *#P*<0.05, ##*P*<0.01, ###*P*<0.001, n = 6).

An important component of the allergic asthma model is the production of IgE. Therefore, serum IgE levels were measured from CKA-challenged mice or PBS, MK and PPY-treated groups. Serum IgE levels from CKA-induced asthmatic mice were significantly increased when compared with the PBS-treated NC group (PBS only), but PPY (1, 2, 10 mg/kg) -treated mice had significantly decreased levels of IgE (Figure 4).

### Effect of PPY on total cell infiltrate and inflammatory cells in the BAL fluid

**Figure 5:**
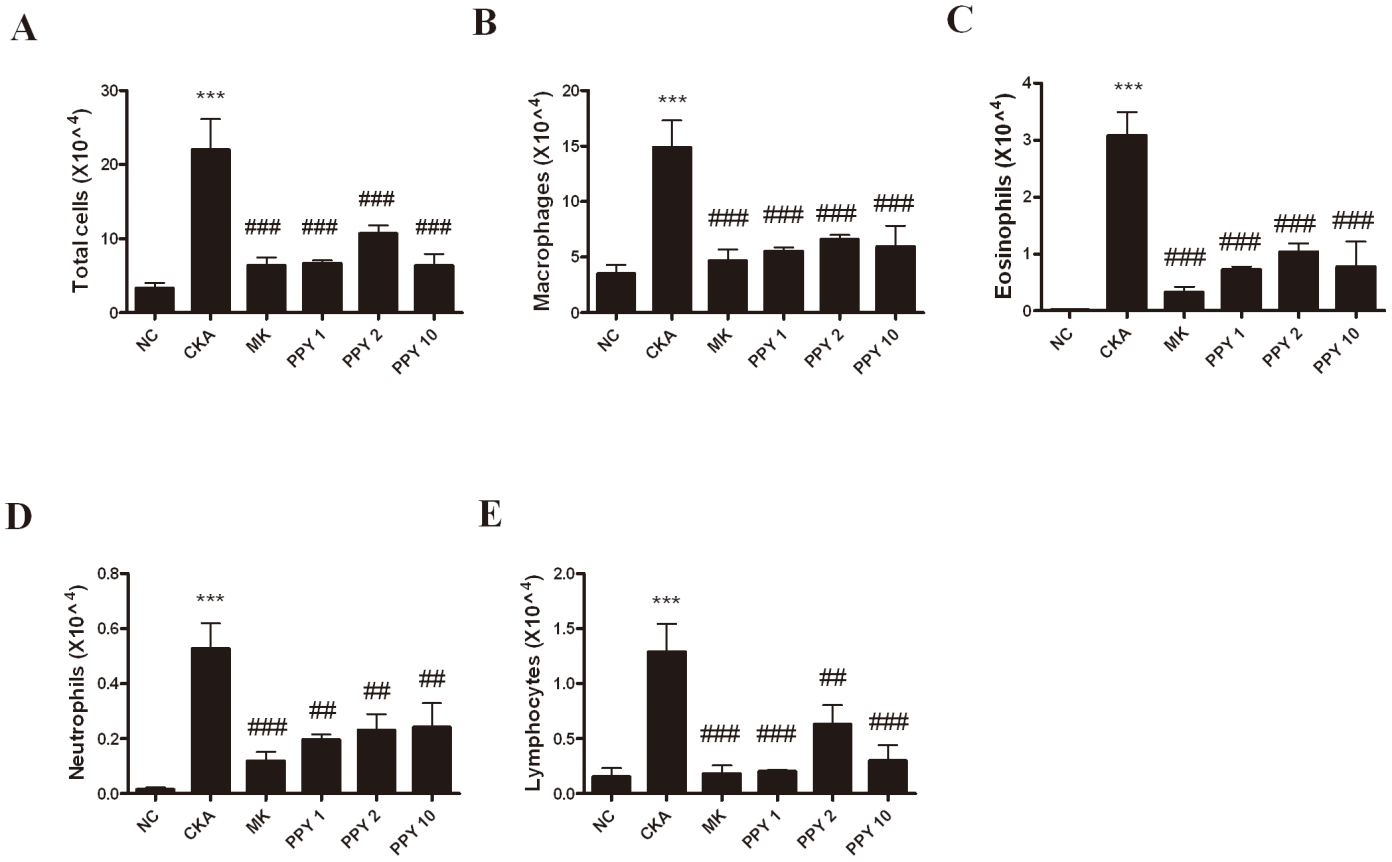
Inflammatory cell accumulation in the BAL fluid. BAL fluid was obtained from the lungs of mice following the induction of allergic asthma. Cells were isolated by centrifugation and stained with Diff-Quik stain reagent. Cell numbers were determined using a light microscope to count cells in at least five squaresof a hemocytometer after excluding dead cells using Trypan blue. Total leukocytes (A), macrophages (B), eosinophils (C), neutrophils (D) and lymphocytes (E) from the BAL fluid were counted. Normal control mice treated with PBS only (NC), CKA-challenged mice treated with PBS (CKA), CKA-challenged mice treated with 10 mg/kg of Montelukast (MK), CKA-challenged mice treated with 1 mg/kg of PPY (PPY 1), CKA-challenged mice treated with 2 mg/kg of PPY (PPY 2) and CKA-challenged mice treated with 10 mg/kg of PPY (PPY 10). The data are shown as the mean ± S.E.M. Statistical analysis was conducted by one-way ANOVA followed by the Newman-Keuls Multiple Comparison test (significantly different from NC, *P<0.05, **P<0.01, ***P<0.001; significantly different from CKA, *#P*<0.05, ##*P*<0.01, ###*P*<0.001, n = 6).

CKA sensitization and challenge significantly increased the total number of cells in the BAL fluid, significantly increasing the macrophage, eosinophil, neutrophil and lymphocyte populations. Treatment groups with PPY (1, 2, 10 mg/kg) or MK group show remarkable decrease in the total numbers of leukocytes, eosinophils, neutrophils, lymphocytes and macrophages in BAL fluid compared to the CKA group (Figure 5).

### Effect of PPY administration on lung histology in CKA-challenged mice

**Figure 6:**
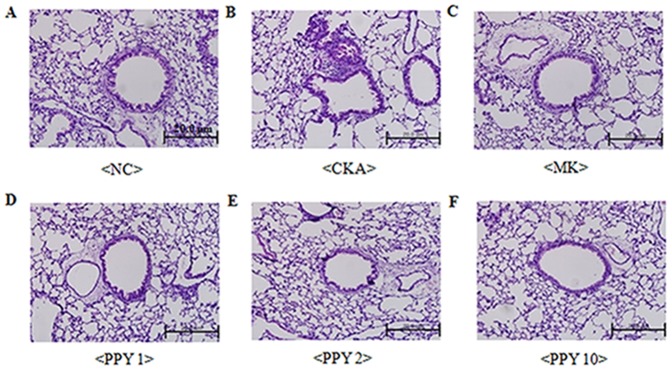
The effect of PPY treatment on airway inflammation in CKA-sensitized mice. Balb/c mice were sensitized and challenged with CKA. Lung sections were fixed, sectioned at 4 µm thickness, and stained with hematoxylin and eosin and then examined by light microscopy (magnification ×200). A) normal control mice treated with PBS only (NC), B) CKA-challenged mice treated with PBS (CKA), C) CKA-challenged mice treated with 10 mg/kg of Montelukast (MK), D) CKA-challenged mice treated with 1 mg/kg of PPY (PPY1), E) CKA-challenged mice treated with 2 mg/kg of PPY (PPY 2), F) CKA-challenged mice treated with 10 mg/kg of PPY (PPY 10).

**Figure 7:**
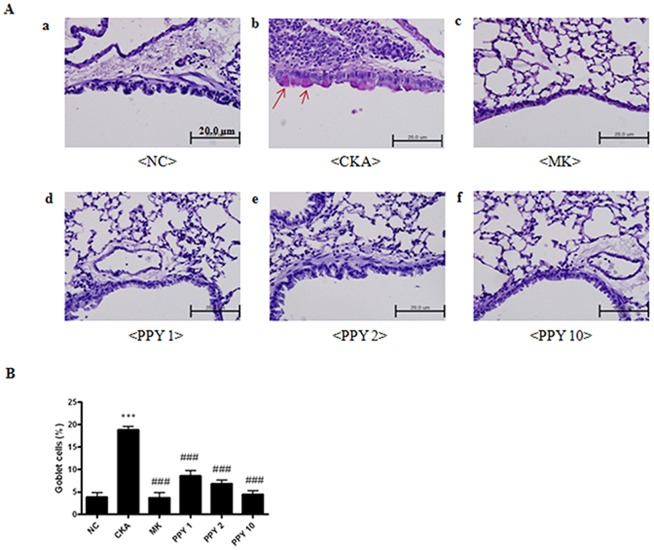
The effect of PPY treatment on the histopathologic changes in lung tissues during CKA-induced allergic asthma mice. (A) Histological examination of mucus secretion in lung tissue. Lung tissue was fixed, sectioned at 4 µm thickness, and stained with periodic acid Schiff (PAS) for mucus production (magnification ×400). A) normal control mice treated with PBS only (NC), B) CKA-challenged mice treated with PBS (CKA), C) CKA-challenged mice treated with 10 mg/kg of Montelukast (MK), D) CKA-challenged mice treated with 1 mg/kg of PPY (PPY1), E) CKA-challenged mice treated with 2 mg/kg of PPY (PPY 2), F) CKA-challenged mice treated with 10 mg/kg of PPY (PPY 10). (B) To aid interpretation, quantified as the percentage of positive goblet cells. The data are shown as the mean ± S.E.M. Statistical analysis was conducted by one-way ANOVA followed by the Newman-Keuls Multiple Comparison test (significantly different from NC, *P<0.05, **P<0.01, ***P<0.001; significantly different from CKA, *#P<0.05, ##P<0.01, ###P<0.001*, n = 6).

**Figure 8:**
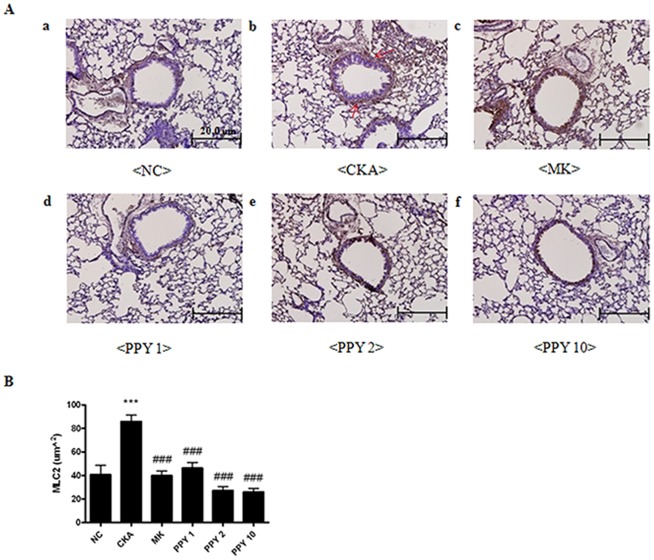
The effects of PPY treatment on airway remodeling in lung tissue, MLC2 immunohistochemistry. (A) Representative immunohistochemistry of the smooth muscle myosin light chain 2 expression in a subject with allergic inflammation and enlarged view of a smooth muscle bundle. The arrows indicate MCL2 positive cells (brown color indicates positivity, magnification ×200). A) normal control mice treated with PBS (NC), B) CKA-challenged mice treated with PBS (CKA), C) CKA-challenged mice treated with 10 mg/kg of Montelukast (MK), D) CKA-challenged mice treated with 1 mg/kg of PPY (PPY1), E) CKA-challenged mice treated with 2 mg/kg of PPY (PPY 2), F) CKA-challenged mice treated with 10 mg/kg of PPY (PPY 10). (B) To aid interpretation, the thickness of smooth muscle was calculated based on the immunohistochemical images. The areas of bronchial smooth muscle were measured using Image-Pro Plus software 6.1 (Media Cybernetics, Warrendale, PA, U.S.A) at magnifications of 200×. The data are shown as the mean ± S.E.M. Statistical analysis was conducted by one-way ANOVA followed by the Newman-Keuls Multiple Comparison test (significantly different from NC, *P<0.05, **P<0.01, ***P<0.001; significantly different from CKA, *#P<0.05, ##P<0.01, ###P<0.001*, n = 6).

Lung tissues were stained with H&E, PAS and MLC2 to analyze the effects of PPY on the histological features of asthma. Histological sections of lung tissue from mice that were exposed to CKA exhibited airway inflammation and were found to have infiltrating eosinophils in the peribronchial regions of the lung. Conversely, PPY (1, 2, 10 mg/kg) significantly decreased this eositophil-rich inflammatory cell infiltration compared with CKA exposed group (Figure 6). Goblet cell hyperplasia is common features of asthmatic airways [41]. We next evaluated airway goblet cells and mucus with Periodic acid Schiff (PAS) stained. PAS-positive mucus secreting goblet cells around the bronchial airway were detected in the CKA exposed group. On the other hand, PPY (1, 2, 10 mg/kg) treatment substantially decreased PAS-positive goblet cells around the bronchial airway (Figure 7). CKA-challenged mice showed expression of myosin light chain 2 (MLC2) in the peribronchial muscle layer of the lung, and PPY (1, 2, 10 mg/kg) treatment abrogated the expression of this protein (Figure 8). These findings demonstrate that PPY has the potential to counteract allergic asthma-associated airway inflammation and remodeling.

## Discussion

Montelukast (trade names Singulair, Montelo-10, and Monteflo and Lukotas in India) is a leukotriene receptor antagonist (LTRA) used for the maintenance treatment of asthma to relieve symptoms of seasonal allergies [42], [43]. However, Montelukast has side effects such as regarding anxiety and other neuropsychiatric adverse events [44]. There is a need for the development of alternatives which is safe and efficacious treatments for allergic asthma. In mechanistic view, the action of Montelukast was mainly restricted to the respiratory mucosal areas, whereas corticosteroid affects not only immune cells but also in variety cells throughout the whole body systems. Since the main target for PPY was believed to be lung epithelial cells [22], it is more reasonably to compare the action of PPY with Montelukast than corticosteroid in this study.

Asthma is a clinical complication of persistent airway inflammation and subsequent airway hyper-responsiveness, and it is a leading cause of morbidity and mortality in critically ill patients. Understanding the pathogenesis and mechanisms of allergic asthma is important for finding appropriate therapeutic targets so that patients can receive symptomatic treatment [45]. Although there are treatments that are currently available for the management of asthma targeted at dampening airway inflammation and relaxing airway smooth muscles, including inhaled corticosteroids and β_2_ agonists, they do not eliminate the occurrence of disease because they do not alter the underlying pathology [46]. Moreover, inhaled corticosteroids have several local and systemic side effects including, oropharyngeal candidiasis, dysphonia, lingual hypertrophy, pharyngitis, growth failure, bone metabolism disorders, adrenal suppression and glaucoma, especially if used with large doses and for a long time [47]. These facts highlight the need for novel molecules that have fewer side effects, preferably of natural origin, for the treatment of allergic asthma.

Cockroach allergy (CKA) is associated with development of asthma, especially in inner-cities where it affects up to 80% of asthmatic children that are sensitized and exposed to allergens produced by Blattella germanica[48], [49]. Bla g1 and Bla g2 are the main components of CKA allergen. Exposure to Bla g 1 or Bla g 2 causes allergic reaction mediated with IgE [50], [51]. In this study, we used standardized CKA which is quality-controlled by quantitating Bla g1 and Bla g2 (Hollister-Stier, Spokane, WA, U.S.A.) [23].

In a previous study, Lee *et al.* demonstrated that eotaxin is significantly decreased in epithelial cells that have been stimulated with pro-inflammatory cytokines and treated with PPY. We demonstrated that PPY has inhibitory effect on allergic asthma through blocking the activation of the NF-kB and ERK1/2 signaling pathways [22]. These results suggest that PPY has the potential to be used in the treatment of asthma and allergic disease through inhibition of eotaxin (CCL11) secretion and eosinophil migration into the airway epithelium [52], [53].

Therefore, we investigated the anti-inflammatory and immunomodulatory effects of PPY in a mouse model of cockroach allergic inflammation with regard to airway function, antigen-induced inflammatory infiltrates in the airways, local Th2 cytokines and eotaxin (CCL11) production, serum immunoglobulin levels and histopathological changes of lung tissues.

IL-4, IL-5, and IL-13 are key Th2-type cytokines that play important roles in the development of allergic asthmatic responses [54]. Also the level of eotaxin (CCL11) is related to the degree of eosinophilic airway inflammation and sub-epithelial fibrosis. IL-4 promotes the differentiation and proliferation of Th2-type T cells, and the switching of B cells to produce IgE. Blocking IL-4 by using monoclonal antibodies decreased IgE levels and airway eosinophil accumulation in allergic mice [55]–[57]. IL-5 plays an important role in the differentiation, maturation and survival of eosinophils, which leads to infiltration into the airways. Moreover, previous studies have shown that eosinophilic inflammation could not be developed in the absence of IL-5, and it was shown that IL-5 signaling in the airways was necessary for the development of asthma in allergic mice [58], [59]. IL-13 is involved in AHR, promoting IgE isotype switching in B cells and mucus secretion in the airway mucosa. IL-13 knockout mice fail to develop AHR after allergen sensitization and challenge even in the presence of severe airway inflammation [60], [61]. Enhanced pause (Penh) is a dimensionless index generally used to evaluate changes in the shape of the airflow pattern entering and leaving a whole-body flow plethysmograpy as mice breathes. The index is sensitive to alterations in the distribution of area under the waveform during exhalation and increases in a non-linear fashion as the normalized area increases near the start of the curve. Penh has been used to evaluate changes in lung function and as a method to evaluate airway reactivity in many other studies [62]. However, Penh simply does not contain the information required to provide a valid estimate of lung mechanics, because it is based on only a single time-varying signal, the pressure inside a plethysmograpy. Penh represents some kind of nonspecific reflection of the breathing pattern [63], [64]. In the present study, PPY (1, 2, 10 mg/kg) treatment significantly reduced the total number of leukocytes and the influx of macrophages, eosinophils, neutrophils, and lymphocytes as control group which was exposed-CKA. The levels of eotaxin (CCL11) and Th2 cytokines such as IL-4, IL-5, and IL-13 were markedly reduced. Serum IgE concentration was also significantly decreased following PPY treatment. These results revealed that the anti-inflammatory effect of PPY can be attributed to the suppression of pro-inflammatory cytokine production in the lung.

Treatment with 10 mg/kg of PPY shows inhibitory effects on CKA induced allergic asthma in mice better than 1 mg/kg of PPY in several biomarkers. For example, 10 mg/kg of PPY treatment significantly reduced the increased Penh values of the CKA-exposed mice at 50 mg/ml of methacholine, but, treatment with 1 mg/kg of PPY group did not. In addition, data from treatment with 1 mg/kg of PPY data did not show significant decrease of IL-13 levels no more than CKA-exposed group. Also, IL-5 and IL-13 levels are remarkably reduced in group of treatment with 10 mg/kg of PPY, comparing to group of treatment with 1 mg/kg of PPY. These results suggest that treatment with 10 mg/kg of PPY is generally more effective than 1 mg/kg PPY in CKA induced allergic asthma.

Airway remodeling, which is composed of pathological changes that occur in asthmatic patients, includes goblet cell hyperplasia, an increased peribronchial muscle layer, angiogenesis and fibrosis. The functional consequences of airway remodeling contribute to an increased susceptibility to asthma exacerbation [65]. Additionally, goblet cell hyperplasia is another cause of asthma airway remodeling that leads to obstruction of the airway through excessive mucus production [66]. Mucus hyper-production by hyperplastic goblet cells is also associated with airway inflammation in asthma and causes airway mucous plugging [67]. Mucous plugging is a primary factor that contributes to mortality rates, and it is associated with severe acute asthma [68], [69]. Increased amounts of MLC2, the central regulator of cellular contraction, have been found in the peribronchial muscle layer from asthmatics [70].

We have shown that PPY had effect on the histological changes of the lung tissue. PPY decreased infiltrating eosinophils in the peribronchial regions and PAS-positive goblet cells around the bronchial airway. Also, expression of myosin light chain 2 (MLC2) in the peribronchial muscle layer of the lung was abrogated. These findings demonstrate that PPY can be a potential treatment to counteract the allergic asthma-associated airway remodeling. In a previous study, Lee *et al.* showed that intraperitoneal administration of PPY exerts profound inhibitory effects on the accumulation of eosinophils in the airways while also reducing the levels of IL-4, IL-5, and IL-13 in the BAL fluid in an ovalbumin-induced asthma model [22]. However, to be a good drug candidate, it is necessary to test the efficacy of PPY in an oral route.

In summary, our investigation demonstrated that PPY inhibited the development of asthmatic inflammation in a cockroach allergen induced airway responses in mice. PPY was as effective as positive control (MK) in most experiments. In this study, it provides reasonable evidences that PPY can be developed as an alternative medication for clinical use.
